# Effects of Genetic Mutations and Chemical Exposures on *Caenorhabditis elegans* Feeding: Evaluation of a Novel, High-Throughput Screening Assay

**DOI:** 10.1371/journal.pone.0001259

**Published:** 2007-12-05

**Authors:** Windy A. Boyd, Sandra J. McBride, Jonathan H. Freedman

**Affiliations:** 1 Laboratory of Molecular Toxicology, National Toxicology Program, National Institute of Environmental Health Sciences, National Institutes of Health, Research Triangle Park, North Carolina, United States of America; 2 Nicholas School of the Environment and Earth Sciences, Duke University, Durham, North Carolina, United States of America; University of Maryland, United States of America

## Abstract

**Background:**

Government agencies have defined a need to reduce, refine or replace current mammalian-based bioassays with testing methods that use alternative species. Invertebrate species, such as *Caenorhabditis elegans*, provide an attractive option because of their short life cycles, inexpensive maintenance, and high degree of evolutionary conservation with higher eukaryotes. The *C. elegans* pharynx is a favorable model for studying neuromuscular function, and the effects of chemicals on neuromuscular activity, i.e., feeding. Current feeding methodologies, however, are labor intensive and only semi-quantitative.

**Methodology/Principal Findings:**

Here a high-throughput assay is described that uses flow cytometry to measure *C. elegans* feeding by determining the size and intestinal fluorescence of hundreds of nematodes after exposure to fluorescent-labeled microspheres. This assay was validated by quantifying fluorescence in feeding-defective *C. elegans* (*eat* mutants), and by exposing wild-type nematodes to the neuroactive compounds, serotonin and arecoline. The *eat* mutations previously determined to cause slow pumping rates exhibited the lowest feeding levels with our assay. Concentration-dependent increases in feeding levels after serotonin exposures were dependent on food availability, while feeding levels decreased in arecoline-exposed nematodes regardless of the presence of food. The effects of the environmental contaminants, cadmium chloride and chlorpyrifos, on wild-type *C. elegans* feeding were then used to demonstrate an application of the feeding assay. Cadmium exposures above 200 µM led to a sharp drop in feeding levels. Feeding of chlorpyrifos-exposed nematodes decreased in a concentration-dependent fashion with an EC_50_ of 2 µM.

**Conclusions/Significance:**

The *C. elegans* fluorescence microsphere feeding assay is a rapid, reliable method for the assessment of neurotoxic effects of pharmaceutical drugs, industrial chemicals or environmental agents. This assay may also be applicable to large scale genetic or RNAi screens used to identify genes that are necessary for the development or function of the pharynx or other neuromuscular systems.

## Introduction

Large numbers of chemicals are produced annually and, without efficient models for toxicological testing, the processes of drug discovery and chemical safety evaluation are often delayed. For many years, government and industry have primarily relied upon mammalian systems to evaluate chemical toxicity. Now, there is a concerted effort to reduce, replace, or refine the use of mammals in laboratory testing [1]. The development of better molecular and computational tools has provided an opportunity for the creation of faster screens using alternative species. Testing with the nematode *Caenorhabditis elegans* offers a unique approach that possesses advantages over cell-based *in vitro* assays, which are rapid but may not be physiologically relevant, as well as mammalian studies, which are slow and expensive but may better represent human responses. *C. elegans* is an excellent alternative model organism for toxicological screening for several reasons. It is simple enough to use in a manner similar to cultured cells, but is a multi-cellular organism that exhibits complex behaviors [2]. There is a high degree of evolutionary conservation between *C. elegans* and higher organisms in many of the stress-response genes and cognate signal transduction pathways that are affected by toxicant exposure [3]. Additionally, *C. elegans* can grow and reproduce in multi-well plates, making it applicable for high-throughput robotic technologies.

For over 30 years feeding behavior and physiology have been studied in *C. elegans* [4]. The *C. elegans* pharynx has been used as a model of cardiac and skeletal muscle cell function and behavior. Rhythmic contractions of the pharynx pump liquid and bacterial food in through the mouth, concentrate the bacteria and expel the excess liquid back out of the mouth, while simultaneously pumping the bacteria backwards into the intestine [5]. This pumping activity is regulated by both internal and external cues, which can be manipulated by exogenous exposures or genetic mutations [6]–[8]. Genetic mutations in *C. elegans* have been described in which feeding rates decrease, or there is irregular transport of bacteria through the pharynx [9].

Traditional methods to measure *C. elegans* feeding were designed to investigate different aspects of the pharyngeal pumping process. Most commonly, pumping rates are counted from visual observations that allow for the surveillance of bulb contractions and abnormal muscle actions [9]–[13]. Attempts to monitor the movement of bacteria through the digestive system led researchers to introduce tracers, such as mineral oil or pigments, into the liquid media [14]. As the fluid dynamics of feeding were elucidated, questions pertaining to the amount of bacteria ingested remained. Therefore, more quantitative methods were developed, including exposing nematodes to iron particles or fluorescent microspheres, and then counting the number of particles in the nematode intestine [15]–[17]. Early studies indicated that uptake of particles was related to pharyngeal pumping rate and nutritional status [18]. Another method, the electropharyngeogram, was designed to measure action potentials of the pharyngeal muscles in live nematodes or dissected pharynxes [6], [19], [20]. However, existing methods for feeding measurement allow observation of only a limited number of nematodes. Moreover, they can be time-intensive and subjective, and may require specialized technical experience.

Here we describe an automated and quantitative method for measuring *C. elegans* feeding that increases the level of throughput such that the feeding behaviors of hundreds of individual nematodes are quantified in minutes. This assay utilizes the COPAS Biosort, an automated nematode analysis, sorting, and dispensing platform [21]–[24], that quantitatively measures feeding using flow cytometry to measure intestinal fluorescence in nematodes after exposure to fluorescent-labeled microspheres.

We validate the assay by comparing the feeding behavior of wild-type *C. elegans* to Eat mutants with known defects in feeding behavior [9], as well as by exposing nematodes to chemicals known to stimulate pumping (serotonin) or uncouple pumping (arecoline). Following validation, two environmental toxicants (cadmium and chlorpyrifos) were used at sub-lethal concentrations to demonstrate the application of the feeding assay as a toxicological screen. Cadmium was chosen as a model toxicant because the effects of exposure have been described using several feeding assays [17], [25], [26]. Chlorpyrifos is an organophosphate pesticide known to cause neurological effects in humans as well as behavioral effects in *C. elegans* [27].

We show that this high-throughput screening technology is a powerful tool for chemical screening using a whole-organism, *in vivo* model. In addition, this technology may be applicable to genetic screens, including genome-wide RNAi or feeding-defective mutant identification.

## Methods

### Nematode culture

All strains of *C. elegans* were obtained from the *Caenorhabditis* Genetic Center (Minneapolis, MN), and were maintained at 20°C on K-agar plates seeded with *E. coli* OP50 [28], [29]. The Bristol N2 strain of *C. elegans* was used as the wild-type. The following strains were used to investigate feeding in mutants previously reported to express varying degrees of Eat phenotypes: DA531 *eat-1(ad427)*, DA453 *eat-2(ad453)*, DA465 *eat-2(ad465)*, DA1113 *eat-2(ad1113)*, DA1116 *eat-2(ad1116)*, DA819 *eat-4(ad819)*, DA467 *eat-6(ad467)*, DA792 *eat-6(ad792)*, DA521 *eat-7(ad450)*, DA599 *eat-8(ad599)*, DA606 *eat-10(ad606)*, DA522 *eat-13(ad522)*, DA573 *eat-14(ad573)*, DA602 *eat-15(ad602)*, JT609 *eat-16(sa609)*, DA707 *eat-17(ad707)*, ST6 *eat-20(nc-4)*, and DA695 *egl-19(ad695)*. *C. elegans* eggs were prepared as previously described to yield age-synchronized adult nematodes [30].

### Feeding assay

Nematodes were transferred to the sample cup of the COPAS Biosort [31] (Union Biometrica Inc., Somerville, MA, USA) and diluted to approximately 1 nematode/µL. The COPAS Biosort was used to sort nematodes based on two size characteristics, their length or “time of flight” (TOF) and optical density or “extinction” (EXT), as well as fluorescence. Twenty-five adults were then dispensed into each well of a 96-well plate, containing a mixture of K-medium [25], chemical (if tested), and *E. coli* at a final volume of 50 µL. Stock solutions of arecoline hydrobromate, serotonin creatinine sulfate complex, and cadmium chloride (Sigma Chemical Co., St Louis, MO) were prepared in K-medium. Chlorpyrifos stocks (Columbus, OH) were prepared in DMSO and all groups (including controls) were tested at final concentrations of 1% DMSO, a concentration found not to affect nematode feeding (data not shown). To ensure that the same concentrations of bacteria were present at the start of each experiment and to determine the approximate amount of bacteria eaten over exposure periods, the level of *E. coli* in each well was assessed by determining the optical density at 550 nm immediately after nematode transfer, and after 4- or 24-h incubations.

Following incubation for 4 h (serotonin, arecoline) or 24 h (*eat* mutants, cadmium, chlorpyrifos), 5 µL aliquots of Fluoresbrite® polychromatic red microspheres (0.5 µm diameter; diluted 1∶20 in K-medium) (Polysciences, Inc., Warrington, PA) were pipetted into each well and rotated on a nutator mixer for 5 min . This size of microsphere was selected because they are similar to the size of *E. coli* that is used as a food source for the nematodes. The nematodes were allowed to ingest the microspheres for 10 additional min (15 min total) and were then anesthetized using 5 µL of sodium azide (10 mM, final concentration), inhibiting further bead ingestion. Nematodes were aspirated from each well with the COPAS Biosort ReFLx; and the length, optical density, and level of fluorescence for individual *C. elegans* were measured.

### Study design, classification of adults and data normalization

A pilot study of the feeding assay using wild-type and four *eat* mutants was conducted to determine the number of experimental replicates required for optimal statistical power. Considerable differences in the center and spread of fluorescence distributions were seen across experimental days, and day-to-day variability comprised a significant amount of overall variability. In addition, distributions of fluorescence showed long right tails. Across all pilot experiments, fluorescence for wild-type nematodes had a median of 1.54, with medians for each of the 23 experimental days ranging from 40% to 160% of this amount. To determine the number of replicates needed for each *eat* mutant strain in the feeding assay, the pilot study data were used in a mixed effects model [32]. Fluorescence was used as the response, mutant types as fixed effects, day as a random effect, and mutant by day interactions as random effects. Based on this model, the variability in the estimate of the mean fluorescence could be reduced by a factor of 25 by repeating the experiment over 3 days rather than 2; repetition of the experiment over 4 days further reduced variability in the estimate of the mean by a factor of 2.1. Based on the results of the pilot study, each measurement was repeated in six wells on each plate, as well as across three (chemical exposure) or four (mutant) experimental days, resulting in a minimum of 600 or 800 nematodes sampled for each experimental condition, respectively.

At the time of measurement (after 4- or 24-h incubations), the nematode population in each well consisted of adults and progeny. Because the feeding assay quantifies feeding in age-synchronized adult nematodes with fully developed nervous systems, it was necessary to separate the adults from their offspring for data analysis. To classify adult and larval stage nematodes based on TOF and EXT measurements, a non-parametric, robust clustering method for large datasets was used. The program CLARA (Clustering Large Applications) [33] is based on k-medoid partitioning, in which a search is made for a user-specified number of representative objects or medoids (in this case, representative measurements of TOF and EXT) in the dataset. CLARA is less affected by outliers than the well-known k-means clustering algorithm. In the application presented here, two clusters are specified *a priori*, representing adults and larvae. The optimal clustering configuration minimizes the average Euclidean distance between each element of the dataset and its representative object.

Once adults were classified using the CLARA algorithm, a normalization procedure was used on the fluorescence data to account for the effects of nematode size on fluorescence, skewed fluorescence distributions, and day-to-day variability. To account for the effect of nematode size on fluorescence and adjust for any possible relationship between gut volume and nematode size, each fluorescence measurement was “size-adjusted” by dividing by its respective length or TOF measurement. Because considerable differences in the center and spread of size-adjusted fluorescence (fluorescence/TOF) distributions were seen across experimental days (data not shown), further normalization of size-adjusted fluorescence measurements was necessary prior to statistical analysis and testing. A square root transformation was applied to the wild-type *C. elegans* size-adjusted fluorescence measurements to eliminate long right tails and make each day's measurements approximately normally distributed. To aggregate fluorescence data across experiments and adjust for day-to-day variability, size-adjusted and square-root transformed fluorescence measurements for each mutant and each day were then centered and scaled according to the mean and variance of that day's wild-type nematode size-adjusted and square-root transformed fluorescence measurements. We refer to measurements of fluorescence that have been size-adjusted, square-root transformed, and centered and scaled to their respective day's wild-type *C. elegans* measurements as “normalized fluorescence” measurements.

### Ranking the impacts of eat mutations on feeding

To test differences in feeding between and among adult strains, as well as between chemical exposures, a bootstrap procedure was used to estimate means of normalized fluorescence measurements for each strain as well as bootstrap standard errors and 95% bootstrap percentile intervals for the mean. The bootstrap procedure was as follows: (1) For each experimental day, plate and mutant, repeat the following procedure 500 times: (a) Draw a bootstrap sample of size-adjusted, square root transformed fluorescence measurements for wild-type nematodes of sample size equal to 75% of the total number of nematodes measured for that day:plate combination. Bootstrap samples were drawn from observed wild-type sample sizes ranging from 81 to 217 with a mean of 179. From each bootstrap sample, calculate the wild-type *C. elegans* sample mean and standard deviation. (b) For each mutant strain, draw a bootstrap sample of size-adjusted, square-root transformed fluorescence measurements of sample size equal to 75% of the total number measured for that day: plate combination. Observed sample sizes depended on the mutant and ranged from 81 to 203 with a mean of 157. Center and scale each mutant's size adjusted and square-root transformed fluorescence measurement to its respective day's and plate's wild-type readings by subtracting the wild-type sample mean and dividing by the wild-type sample standard deviation from step (a). (c) Calculate the mean of the centered and scaled mutant measurements in (b). This represents a single bootstrap sample of the mean normalized fluorescence measurement for a given mutant. For each mutant, 500 bootstrap means were calculated.

Because the sampling distributions of bootstrap means for each mutant appeared approximately normal with approximately equal variance, the Tukey-Kramer multiple comparison procedure was used to rank the bootstrap means of normalized fluorescence for each mutant by degree of feeding reduction. The critical value for the studentized range distribution at α = 0.05, based on 19 and 9481 degrees of freedom, was q(19,∞) = 4.97.

### Quantifying the effects of chemical compounds

For exposures to neuroactive compounds (serotonin and arecoline) as well as environmental contaminants (cadmium and chlorpyrifos), adult nematodes were identified using the CLARA [33] clustering algorithm, and adult fluorescence measurements were normalized as described above for statistical analysis. That is, size-adjusted, square-root transformed fluorescence measurements for each chemical concentration, feeding status and day were normalized with respect to their respective day's control nematode measurements. Bootstrap procedures identical to those described above for *eat* mutants were used to calculate bootstrap means, standard errors and 95% bootstrap percentile intervals for the mean under each chemical and food condition.

Of interest in the analysis of exposures to the neuroactive chemicals arecoline and serotonin were statistical differences in feeding levels between chemical exposures and their respective controls, and a Dunnett procedure was used in these cases to perform multiple comparisons. Examination of the sampling distributions of bootstrap means for nematodes indicated that distributions could reasonably be assumed to be approximately normal with approximately equal variance, allowing for two-sample t-tests between controls and each chemical/food condition. The critical value of *t* for the Dunnett procedure is approximately 3.06, based on 5 treatments and 1096 degrees of freedom (500 bootstrap samples per mean, with 6 means per combination of chemical and food including the control mean).

For nematode exposures to the environmental contaminants cadmium and chlorpyrifos, concentration-response curves were fit to the size-adjusted fluorescence at each concentration level. A four-parameter sigmoidal growth model derived from steady-state catalytic kinetics [34] was applied. The model, known as the Morgan-Mercer-Flodin model [35] was parameterized as follows:
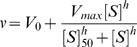



Here, *v* is taken to be the feeding level at chemical concentration [*S*]. *V_0_* is the feeding level at the concentration producing the maximal effect. *V_max_* is the maximal effect of the chemical over the concentration range. [*S*]_50_ is the concentration of the chemical that produces 50% of the normalized fluorescence level in the absence of the chemical, commonly known as the EC_50_. *h* is known as the Hill coefficient. When *V_0_* = 0 and *h* = 1, the expression reduces to the Michaelis-Menton model. When *V_0_* = 0, the expression reduces to the Hill model. A non-zero value of *V_0_* allows for a non-zero y-intercept. Parameters were estimated using a quasi-Newton method with positivity constraints [36] to minimize the sum of squared differences between observed and fitted values.

## Results

**Figure 1:**
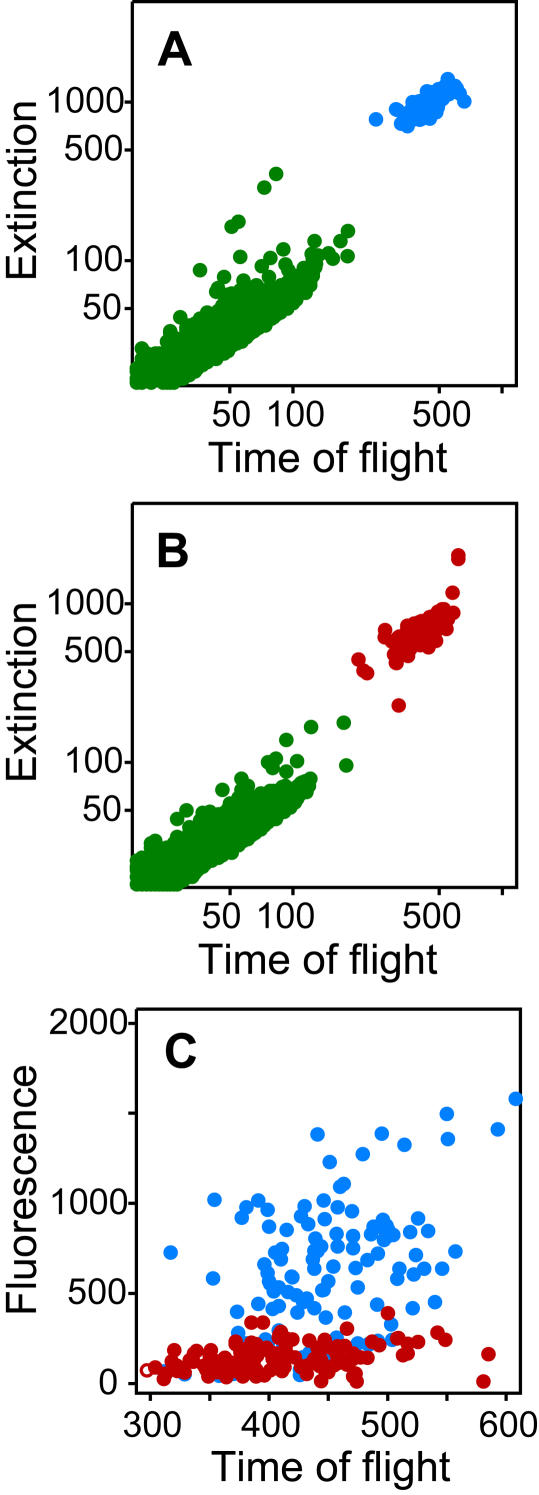
Separation of adult *C. elegans* from their offspring and corresponding adult feeding. Extinction (optical density; log_10_ scale) versus time of flight (length; log_10_ scale) for (*panel A*) wild-type and (*panel B*) *eat-2(ad465)* nematodes, showing the results of the clustering algorithm to separate either wild-type (*blue)* or *eat-2* (*red)* adults from larvae (*green*). In *panel C* the relationship between fluorescence (accumulated microspheres) versus time of flight for the adult wild-type (*blue*) and *eat-2* (*red*) nematodes is shown. Each point corresponds to a single nematode.

The assay quantifies feeding in age-synchronized adult nematodes with fully developed nervous systems, and thus, it was necessary to separate the adults from their offspring. Plots of nematode EXT versus TOF showed clear separation between two clusters of points corresponding to adults and offspring (Fig. 1, A and B). To differentiate between adults and their offspring, a statistical clustering algorithm, CLARA, was applied to measurements of TOF and EXT. The resulting classifications are shown in Figure 1 for two *C. elegans* strains, wild-type and *eat-2*(*ad465*). Overall, statistical classification of measurements as adults or offspring using CLARA corresponded well with visual inspection of clusters of points in plots of TOF versus EXT for all experiments. The excellent performance of the algorithm across all experiments, both for mutant strains and chemical exposures, was due to the clear separation of adults and offspring at the time point measured; that is, there were no worms present at intermediate larval stages between L1 and adult. Of the adult nematodes originally loaded (either by mutant strain or chemical treatment) the clustering algorithm classified approximately 82%. Differences between the number of adults loaded on plates and the number classified as adults can be primarily attributed to the technical limitations of the COPAS ReFLx Sampler aspirating tool, whose manufacturer reports ∼85% recovery rate (Union Biometrica, personal communication). Figure 1C shows the relationship between fluorescence and TOF in adult nematodes for the two nematode strains. Fluorescence measurements of nematodes classified as adults were normalized as previously described and used to quantify feeding.

### Ranking the impacts of *eat* mutations on feeding

**Figure 2:**
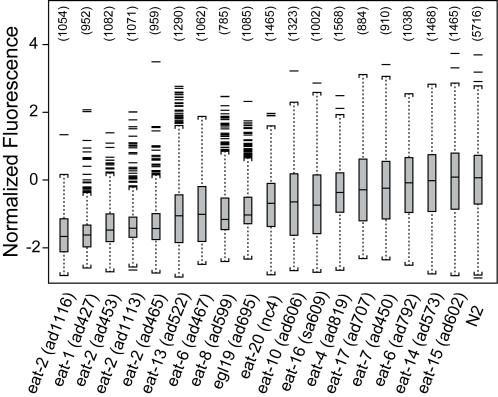
Comparison of wild-type and *eat* mutant *C. elegans* feeding. Boxplots of normalized fluorescence for 19 strains of adult nematodes aggregated over all experiments. Normalization of fluorescence measurements consisted of size-adjustment, square-root transformation, and centering and scaling to the respective day's wild-type measurements. The upper and lower limits of each box give the 25th and 75th percentiles of the data respectively, and the line inside the box gives the median. Whiskers of the boxplot extend to 1.5 times the interquartile range, with extreme measurements indicated by horizontal lines. Sample sizes are given in parentheses above each boxplot.

Previous descriptions of Eat phenotypes are listed in Table 1, and were based on direct observations of a limited number of individual nematodes. Similar to observations in wild-type feeding, Eat phenotypes are known to vary among individuals with identical genotypes [12]. Feeding mutations are known to result in shorter nematodes [37], which can arbitrarily lead to fluorescence measurements that are lower than those in the longer, wild-type nematodes, regardless of feeding rates. To avoid underestimation of feeding, each fluorescence measurement is size-adjusted by dividing by its respective TOF measurement. Fluorescence measurements are then square-root transformed to achieve approximate normality, and centered and scaled by the mean and variance of their respective day's wild-type *C. elegans* measurements. Thus, nematode feeding is expressed as “normalized fluorescence” (Fig. 2).

**Table 1:** Effects of Eat Mutations on *C. elegans* Feeding

Eat mutation	Feeding Assay Results					Previously Published Descriptions	
	Bootstrap Sampling Results			N a	Degree of Feeding Reduction b	Degree of Eat c	Feeding Phenotype d
	Mean	Std Error	95% CI				
N2	0.00	0.02	−0.04, 0.04	5716	1		Normal
*eat-15(ad602)*	−0.05	0.05	−0.14, 0.05	1465	1	Weak-Medium	slippery pharynx, abnormal bulb contractions
*eat-14(ad573)*	−0.10	0.05	−0.19, 0.00	1471	2	Weak-Medium	abnormal grinder functioning
*eat-6(ad792)*	−0.15	0.06	−0.26, −0.04	1039	2,3	Strong	relaxation-deficient
*egl-4(ad450)* e	−0.19	0.06	−0.30, −0.08	910	3,4	Weak	falls asleep, feeds normally when awake
*eat-17(ad707)*	−0.21	0.07	−0.34, −0.09	884	4	Strong	stuffs corpus and isthmus, abnormal contractions
*eat-4(ad819)*	−0.40	0.05	−0.49, −0.31	1569	5	Weak	abnormal timing of bulb contractions
*eat-16(sa609)*	−0.65	0.05	−0.74, −0.54	1002	6	Strong	slippery pharynx
*eat-10(ad606)*	−0.66	0.05	−0.76, −0.55	1323	6	Strong	slippery pharynx
*eat-20(nc-4)* f	−0.74	0.05	−0.82, −0.65	1465	7	Weak-Medium	slow pumping, slippery pharynx
*egl-19(ad695)* g	−0.85	0.05	−0.95, −0.75	1085	8	Weak	relaxation-deficient
*eat-8(ad599)*	−0.91	0.06	−1.04, −0.78	785	9	Strong	brief, rare pumps
*eat-6(ad467)*	−0.92	0.07	−1.06, −0.80	1062	9	Strong	relaxation-deficient
*eat-13(ad522)*	−0.99	0.06	−1.11, −0.88	1293	10	Strong	slippery pharynx, abnormal bulb contractions
*eat-2(ad465)*	−1.34	0.07	−1.47, −1.21	959	11	Medium-Strong	slow, regular pumping
*eat-2(ad1113)*	−1.36	0.06	−1.48, −1.25	1071	11	Medium-Strong	slow, regular pumping
*eat-2(ad453)*	−1.38	0.06	−1.51, −1.25	1082	11	Medium-Strong	slow, regular pumping
*eat-1(ad427)*	−1.60	0.06	−1.72, −1.49	952	12	Medium-Strong	slow, irregular pumping
*eat-2(ad1116)*	−1.62	0.07	−1.75, −1.49	1054	12	Medium-Strong	slow, regular pumping

a Number of nematodes measured

b Tukey-Kramer multiple comparison procedure, α = 0.05; larger numbers indicate greater reductions in feeding

c Avery www.wormbase.org

d Avery[9] except *eat-20*

e *egl-4* previously called *eat-7*

f From Shibata et al. 2000[10]

g *egl-19* (*ad695*) previously called *eat-1*

To differentiate feeding levels between adult mutant strains, a bootstrap procedure was used to estimate means, standard errors and 95% bootstrap percentile intervals of normalized fluorescence measurements for each strain and a Tukey-Kramer multiple comparison procedure was used to rank the bootstrap means for each strain (Table 1). As expected, results of the bootstrap procedure showed that wild-type and mutant nematodes previously classified with a weak Eat phenotype (e.g. *eat-15*) fed the most over all experimental days, while mutants known to exhibit slow pumping rates (e.g. *eat-1* and *eat-2*) fed the least (Table 1; Fig. 2). Our assay was also able to differentiate between feeding levels of different alleles of the same gene. For example, *eat-2* (*ad1116*), had significantly different feeding measurements than any of the other three *eat-2* alleles, *ad453*, *ad465*, and *ad1113*; while *eat- 6*(*ad467*) was significantly different from *eat-6*(*ad792*).

### Quantifying the effects of neuroactive compounds on feeding

**Figure 3:**
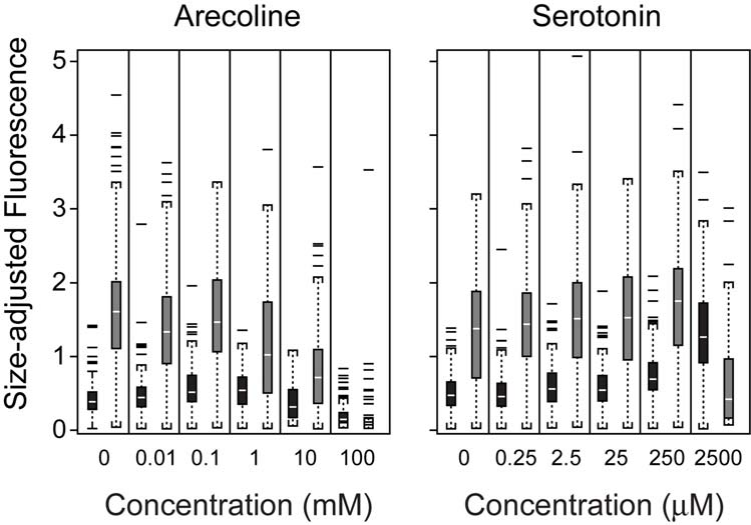
Effects of neuroactive compounds and food availability on wild-type *C. elegans* feeding. Size-adjusted fluorescence values for arecoline (*left panel*) and serotonin (*right panel*) for one representative experiment in the absence (*black*) or presence (*gray*) of bacteria. Each boxplot represents between 250–300 nematodes.

The feeding assay was valuable for screening the effects of chemicals on *C. elegans* neuromuscular activity. Two well-studied chemicals, serotonin and arecoline, were chosen to validate our assay because of their effects on *C. elegans* pharyngeal pumping [13], [15]. Adult nematodes were exposed to arecoline or serotonin after being fed or starved for 4 h in liquid culture. Figure 3 presents the results of one representative experiment illustrating that fed nematodes not exposed to serotonin or arecoline accumulated higher levels of fluorescence than nematodes that were starved for 4 h. Analysis of normalized data across experiments indicated that feeding decreased significantly in arecoline-exposed nematodes in a concentration-dependent fashion starting at 10 mM regardless of feeding status (bootstrap means significantly different from their respective control; α = 0.05, Dunnett multiple comparison *t-*test). In contrast, responses to serotonin exposure were dependent on food availability (Fig. 3). Fluorescence in nematodes exposed to serotonin decreased in fed nematodes exposed to 2500 µM, but increased in starved nematodes exposed to 250 or 2500 µM.

### Application of feeding assay to screening of environmental toxicants

**Figure 4:**
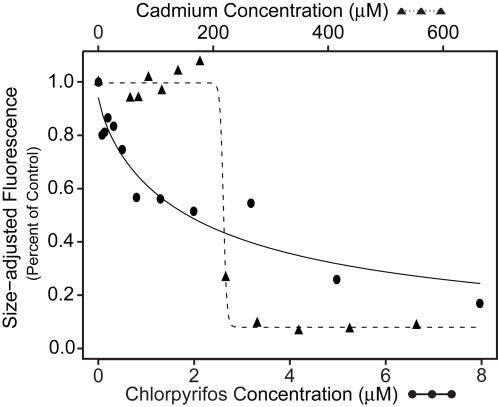
Effect of chlorpyrifos and cadmium on *C. elegans* feeding. Fitted concentration-response curves of single representative experiments based on observed mean size-adjusted fluorescence measurements as a percent of the control for two toxicants, cadmium (*triangles, top axis*) and chlorpyrifos (*circles, bottom axis*). Each point represents approximately 120 nematodes on average, with counts ranging from 104 to 141.

The carcinogenic metal cadmium [38] and the organophosphate insecticide chlorpyrifos [39] were chosen as environmental toxicants to test the applicability of this feeding method for toxicological screening. Figure 4 illustrates representative concentration-response curves for cadmium and chlorpyrifos. Sub-lethal concentrations of both chemicals were tested. Chlorpyrifos was significantly more toxic than cadmium, as evidenced by the calculated concentrations required to reduce nematode feeding by 50% (EC_50_ values) ranging from 1.0 to 2.2 µM and 260 to 324 µM, respectively. In all cases, feeding decreased sharply in nematodes exposed to cadmium concentrations greater than 200 µM, and decreased gradually in nematodes exposed to increasing concentrations of chlorpyrifos (Fig. 4). As evidenced by overlapping confidence intervals, the calculated EC_50_ values were consistent across all repetitions of the experiments for each chemical (Table 2).

**Table 2:** Estimated EC_50_s and 95% maximum likelihood confidence intervals based on 3 repetitions of the *C. elegans* feeding assay.

Chemical	EC_50_ (µM)	95% C.I. * (µM)
Cadmium	262	(226, 299)
	324	(251, 396)
	260	(209, 312)
Chlorpyrifos	1.0	(0.00, 4.6)
	2.2	(0.00, 9.2)
	1.4	(0.00, 4.9)

* Standard errors for EC_50_ values were calculated by fixing other model parameters at their least squares estimates.

## Discussion

A high-throughput, *in vivo* assay that quantitatively measures the activity of the *C. elegans* pharynx is presented. This assay was validated using strains of *C. elegans* that have mutations that affect pharyngeal function, chemicals known to modulate feeding behavior, and environmentally relevant toxicants. In all cases, the results from this study are consistent with previous observations.

There is good agreement between the rankings of the Eat phenotypes measured using the current assay, compared to previously published observations (Table 1). There are, however, exceptions that may be attributed to differences in experimental procedure and interpretation. The original descriptions of the Eat phenotypes were based on direct observations of pharyngeal pumping [9]. These observations utilized a small sample size and were subjective. In contrast, the current methodology examines hundreds of nematodes and is quantitative. In addition, the relative strength of a phenotype may not be directly related to the amount of food consumed. For example, *eat-17(ad707)* was previously described as a strong Eat phenotype, but shows one of the highest feeding levels in the current assay. Higher feeding levels would be predicted based on the observation that *eat-17(ad707)* nematodes “stuff their pharynxes” with bacteria. In contrast, *egl-19(ad695)* is described as a weak relaxation-deficient Eat, but fell in the middle of the feeding levels; possibly because the terminal bulb contractions are not timed properly with those in the corpus preventing transport of microspheres into the intestine.

In examining the applicability of this assay in testing pharmaceutical agents, the effects of two drugs, arecoline and serotonin, known to alter pharyngeal activity in *C. elegans* were examined. The presence of food is known to stimulate pumping whereas serotonin can stimulate pumping, even in the absence of food [15]. In contrast, arecoline inhibits pumping, independent of the presence of food [15], [40]. Consistent with these observations, exposure to the muscarinic agonist, arecoline, resulted in a concentration-dependent inhibition of feeding, independent of the starvation status while serotonin stimulated feeding in starved nematodes, but inhibited feeding in fed animals (Fig. 3). Serotonin increased feeding level in a manner similar to previous reports, in which pumping increased from 22 pumps/min in starved control nematodes to 267 pumps/min in nematodes treated with 10 mg/ml (∼25 mM) serotonin [13]. The effective concentrations for serotonin activity differed slightly between the two studies. This can be attributed to the method of exposure; where previous exposures were on agar plates and the current via liquid medium. In the absence of drugs, we observed that fed nematodes ingested more microspheres than starved nematodes (Fig. 3). These observations were consistent with those previously reported, in that well-fed nematodes pump regularly when bacteria are present, while starved animals pump slowly and irregularly in the absence of food [15], [16].

In previous toxicological studies, feeding has been estimated from the amount of bacteria or particles removed from liquid media [25]–[27], [41]. To test the applicability of the new feeding assay in chemical screening, two environmental toxicants chlorpyrifos and cadmium were examined. Chlorpyrifos inhibits acetyl cholinesterase activity, blocking neuronal activity and paralyzing the animal. *C. elegans* express several acetyl cholinesterase genes that are potential targets of chlorpyrifos [42]. Although the effects of chlorpyrifos on *C. elegans* feeding have not been evaluated, other organophosphate pesticides have been shown to inhibit nematode movement [43]. EC_50 _values ranging from 1.0 to 2.2 µM for feeding inhibition by chlorpyrifos in the current study (Table 2) are similar to the EC_50_ of 5 µM previously reported for movement [27], [43].

Cadmium is not generally considered to be a neurotoxicant, but has been shown to cause decreased motor activity in animals [44], [45]. Cadmium has been shown to decrease feeding in *C. elegans* when measured as changes in optical density, bead ingestion, or red acrylic paint uptake [17], [25], [26]. An EC_50_ for feeding inhibition of 128 µM cadmium was found in a previous study by measuring changes in the optical density of the exposure medium [26]. This is in good agreement with the calculated EC_50_s of 260, 262, and 324 µM cadmium determined using the current assay (Table 2). In addition, as previously observed, cadmium concentrations above 200 µM do not fully inhibit feeding, but instead resulted in minimal feeding levels [25].

An application of the microsphere feeding assay that was not explored in this study is the use of multiple fluorescents probes. The COPAS Biosort is able to simultaneously measure two of three available fluorescence emission wavelengths: 500–520, 535–555, or 600–620 nm [31]. Therefore, the accumulation of “red-labeled” fluorescence microspheres can be quantified while at the same time measuring the expression of a green fluorescence protein probe without interference. Routinely a *myo-2*::GFP expressing transgenic strain of *C. elegans* is used in these assays. If the use of red fluorescent markers were necessary, other fluorescent microspheres are available with various excitation and emission characteristics.

The *C. elegans* fluorescence microsphere feeding assay is a rapid, reliable method for the assessment of neurotoxic effects of pharmaceutical drugs, industrial chemicals or environmental agents. This assay may also be applicable in large scale genetic or RNAi screens [46], [47] used to identify genes that are necessary for the development or function of the pharynx or other neuromuscular systems including mammalian cardiac models.
